# Evaluating the Impact of Enhanced Stent Visualization on PCI Outcomes in Older Patients With Multivessel Disease

**DOI:** 10.1002/ccd.70819

**Published:** 2026-08-21

**Authors:** Marta Cocco, Alberto Sarti, Filippo Maria Verardi, Serena Caglioni, Jacopo Farina, Carlo Penzo, Carlo Tumscitz, Matteo Serenelli, Renè Tezze, Anna Cantone, Andrea Erriquez, Gianluca Campo, Simone Biscaglia

**Affiliations:** ^1^ Cardiology Unit Azienda Ospedaliero‐Universitaria di Ferrara Ferrara Italy

**Keywords:** enhanced stent visualization, multivessel disease, myocardial infarction, older adults, percutaneous coronary intervention, stent optimization

## Abstract

**Background:**

Optimal stent expansion is a key determinant of long‐term outcomes after PCI. Intravascular imaging provides the most accurate assessment of stent deployment but remains underused, especially in older patients with MI and multivessel disease. ESV is an angiographic enhancement technology that may help detect gross stent underexpansion when advanced imaging is not feasible.

**Aims:**

To evaluate the procedural and clinical impact of enhanced stent visualization (ESV)‐guided percutaneous coronary intervention (PCI) in older patients with myocardial infarction (MI) and multivessel disease.

**Methods:**

In this prespecified sub‐study of the FIRE trial, we evaluated the impact of ESV use during PCI in older MI patients. Among 1445 enrolled patients, 331 underwent ESV‐guided PCI and 1114 standard angiography‐guided PCI. Procedural optimization triggered by ESV was defined as additional postdilation, extra stent implantation, or use of intravascular imaging. Minimal lumen diameter (MLD) was assessed before and after ESV‐guided optimization. Clinical outcomes were compared using multivariable and competing‐risk adjusted Cox models.

**Results:**

Operator‐driven optimization occurred in 85% of ESV cases, primarily through postdilation (96%). ESV guidance resulted in a significant MLD increase from 2.51 [2.22–2.87] to 2.93 [2.41–3.32] mm (*p* < 0.001), yielding an acute luminal gain of 0.42 mm (95% CI 0.37–0.47). At 3 years, the composite primary endpoint (death, MI, stroke, or ischemia‐driven revascularization) was lower in the ESV group (adjusted HR 0.70, 95% CI 0.54–0.91; *p* = 0.008).

**Conclusions:**

In older MI patients with complex multivessel disease, ESV‐guided PCI was associated with greater procedural optimization, improved acute stent expansion, and lower long‐term adverse events. While not a substitute for intracoronary imaging, ESV represents a pragmatic and valuable adjunct to enhance PCI quality in settings where intravascular imaging is seldom performed.

**Trial Registration:**

ClinicalTrials.gov identifier NCT03772743.

**Abbreviations**
AF: atrial fibrillation
ARC: Academic Research Consortium
BARC: Bleeding Academic Research Consortium
CA‐AKI: contrast‐associated acute kidney injury
CVA: cerebrovascular accident
ESV: enhanced stent visualization
IVUS: intravascular ultrasound
LVEF: left ventricular ejection fraction
MI: myocardial infarction
MLD: minimal lumen diameter
NSTEMI: non‐ST‐segment elevation myocardial infarction
OCT: optical coherence tomography
PAD: peripheral artery disease
PCI: percutaneous coronary intervention
PSM: propensity score matching
STEMI: ST‐segment elevation myocardial infarction

## 1 Introduction

Enhanced stent visualization (ESV) is a simplified imaging technology designed to improve the accuracy of stent implantation during percutaneous coronary intervention (PCI) [1, 2, 3, 4]. By refining standard coronary angiography, ESV provides clearer delineation of coronary lesions, particularly in the presence of heavy calcification, and supports more precise stent positioning [1, 2, 3, 4]. Although its resolution cannot match that of optical coherence tomography (OCT) or intravascular ultrasound (IVUS), ESV reliably identifies major stent‐related issues such as gross underexpansion or suboptimal alignment, offering practical support in real‐world PCI [1, 2, 3, 4]. Older patients with myocardial infarction (MI) and multivessel coronary disease frequently exhibit complex anatomy, including diffuse disease and severe calcifications, which substantially increase the risk of stent underexpansion and malapposition [5, 6]. These procedural limitations are known contributors to adverse outcomes such as restenosis and stent thrombosis [7, 8, 9]. Despite this, the use of high‐resolution intravascular imaging remains remarkably limited in older MI patients, largely due to concerns related to procedural complexity, additional contrast load, cost, and perceived risk in an already fragile population. As a result, operators often rely on angiography alone, even though advanced imaging would represent the ideal approach for optimizing PCI quality in this setting. In contrast, ESV is widely available and frequently employed, yet its clinical value in older MI patients has never been clearly demonstrated. In particular, while ESV is routinely used to support decision‐making around stent expansion, no study to date has established its prognostic relevance in this population. The FIRE (Functional Assessment in Older MI Patients With Multivessel Disease) trial, which enrolled older patients undergoing PCI for MI, offers a unique opportunity to assess the potential role of ESV in this high‐risk group [10, 11, 12, 13, 14]. The CLASSY‐FIRE sub‐study, a prespecified analysis within the trial, specifically evaluates whether ESV can improve procedural assessment and outcomes in patients with complex coronary lesions. This investigation aims to clarify the real‐world utility of ESV and determine whether this commonly used, but insufficiently studied, tool can meaningfully enhance PCI quality and clinical results in older MI patients.

## 2 Methods

The FIRE trial was a multicenter, investigator‐initiated, randomized superiority study designed to compare physiology‐guided complete revascularization with a culprit‐only strategy in older patients presenting with MI and multivessel coronary artery disease [10, 11, 12, 13, 14]. The full trial design, baseline characteristics, and primary results have been reported previously [10, 11, 12, 13, 14]. Eligible participants were adults aged ≥ 75 years admitted with either ST‐segment elevation MI (STEMI) or non‐ST‐segment elevation MI (NSTEMI) who underwent successful PCI of the culprit lesion and exhibited at least one non‐culprit coronary stenosis measuring 50%–99% in a vessel with a reference diameter of ≥ 2.5 mm [10, 11, 12, 13, 14]. Key exclusion criteria included uncertainty regarding culprit‐lesion identification, the presence of non‐culprit disease in the left main coronary artery, planned or prior surgical revascularization, and an estimated life expectancy of less than 1 year [10, 11, 12, 13, 14]. All patients provided written informed consent. The study protocol was approved by the institutional review board at each participating center. The trial was registered at ClinicalTrials.gov (identifier NCT03772743). The corresponding author had full access to the complete data set and is responsible for the accuracy and integrity of the analyses. Data supporting this manuscript are available from the FIRE Study Executive Committee upon reasonable request.

### 2.1 Study Procedures

Patients were randomized between July 18, 2019, and October 25, 2021. In the physiology‐guided complete revascularization arm, all non‐culprit lesions underwent functional evaluation using wire‐based indices—either hyperemic or non‐hyperemic—or angiography‐derived quantitative flow ratio (Medis Medical Imaging Systems, Leiden, The Netherlands) [10, 11, 12, 13, 14]. Lesions identified as functionally significant were subsequently treated with PCI and stent implantation. Conversely, patients allocated to the culprit‐only strategy did not receive any revascularization of non‐culprit lesions, regardless of angiographic severity [10, 11, 12, 13, 14]. In both treatment groups, the use of sirolimus‐eluting, biodegradable‐polymer, ultrathin stents (Supraflex Cruz, Sahajanand Medical Technologies Ltd.) was strongly encouraged. All patients were treated with guideline‐recommended optimal medical therapy throughout the study period.

### 2.2 ESV and Angiographic Core‐Lab

For this analysis, ESV technology (ClearStent, Siemens Healthineers AG, Erlangen, Germany) was available across participating centers, but its use was not prespecified by the study protocol [10, 11, 12, 13, 14]. Deployment of ESV during PCI, on either culprit or non‐culprit lesions, was left entirely to operator discretion and performed according to local practice. All angiographic datasets were analyzed offline by an independent, blinded core laboratory (University of Ferrara, Italy). The core lab adjudicated whether ESV had been used for any treated lesion and classified patients accordingly into an ESV‐guided or non‐ESV‐guided group. Quantitative coronary analyses were performed using QAngio XA software (Medis Medical Imaging Systems, Leiden, the Netherlands). In patients undergoing ESV, the blinded angiographic core laboratory measured quantitative coronary angiography (QCA)‐derived minimal lumen diameter (MLD) on conventional angiographic frames obtained immediately before and after the corrective maneuvers prompted by the qualitative ESV assessment.

### 2.3 Study Endpoints

The first objective of this analysis was to quantify the proportion of patients in whom ESV use prompted further procedural optimization. Such optimization was defined as any operator‐initiated maneuver following ESV assessment, including postdilation (initial or repeated, with a larger balloon or higher inflation pressure), implantation of additional stent(s), or the use of advanced intracoronary imaging (IVUS or OCT) to guide or refine the final result. The second objective was to evaluate changes in MLD measured by means of QCA, before and after ESV‐guided optimization, in order to determine the acute luminal gain attributable to ESV‐driven procedural adjustments. Finally, clinical outcomes were compared between patients undergoing ESV‐guided PCI and those treated with angiography‐guided PCI alone. This comparison was based on the primary composite endpoint of the FIRE trial, consisting of death, MI, stroke, or ischemia‐driven revascularization within 3 years of randomization [10, 11, 12, 13, 14]. Secondary endpoints included the composite of cardiovascular death or MI, the individual components of the primary endpoint, and stent thrombosis. All outcome events were reported by site investigators and adjudicated by an independent clinical events committee blinded to treatment allocation, using Academic Research Consortium (ARC) and Bleeding Academic Research Consortium (BARC) definitions [15, 16].

### 2.4 Statistical Analysis

Normality of continuous variables was assessed using the Shapiro–Wilk test. Continuous variables were reported as mean (SD) or median [IQR], and comparisons were performed using the Student *t*‐test or Wilcoxon rank‐sum test, as appropriate. Categorical variables were summarized as counts and percentages and compared using Pearson's *χ* ^2^ test or Fisher's exact test when required. Time‐to‐event outcomes were analyzed using Kaplan–Meier estimates and Cox proportional‐hazards models, stratifying patients according to whether they underwent ESV‐guided or non‐ESV‐guided PCI. Multivariable models were adjusted for prespecified confounders with established prognostic relevance, including age, sex, diabetes mellitus, prior MI, estimated glomerular filtration rate < 60 mL/min/1.73 m^2^, peripheral artery disease, clinical presentation, Killip class, and culprit vessel (Table 1). The proportional hazards assumption was verified using Schoenfeld residuals and was satisfied for all outcomes (*p* > 0.05). To account for competing risks, Fine–Gray subdistribution hazard models were used. For outcomes including cardiovascular death, non‐cardiovascular death was treated as a competing event. For all other secondary and safety endpoints, overall mortality served as the competing risk. Given baseline imbalances between the ESV‐guided and non‐ESV‐guided groups, a propensity score matching (PSM) analysis was performed as a sensitivity approach. The propensity score was estimated using a logistic regression model incorporating the same covariates used for multivariable adjustment. One‐to‐one nearest‐neighbor matching without replacement was applied, using a caliper of 0.2 SD of the logit of the propensity score. This procedure yielded two smaller but closely comparable cohorts with adequate covariate balance across all matched variables. All patients in the ESV‐guided group were successfully matched to a corresponding patient in the non‐ESV‐guided group. Treatment effects on clinical endpoints were then reassessed within the matched population and reported accordingly. All analyses were conducted using R software (R Foundation for Statistical Computing, Vienna, Austria).

**Table 1:** Baseline characteristics.

Characteristic	No ESV (*n* = 1114)	ESV (*n* = 331)	*p*
Age, years	81 ± 4	80 ± 4	0.019
Female sex, *n* (%)	405 (36.4)	123 (37.2)	0.790
Medical history, *n* (%)			
Hypertension	903 (81.1)	282 (85.2)	0.085
Dyslipidemia	590 (53.0)	169 (51.1)	0.542
Diabetes	361 (32.4)	102 (30.8)	0.591
Current smoker	92 (8.3)	31 (9.4)	0.533
Prior MI	159 (14.3)	61 (18.4)	0.065
Prior PCI	198 (17.8)	59 (17.8)	0.986
History of AF	154 (13.8)	46 (13.9)	0.977
eGFR < 60 mL/min	56.70 (20.5)	57.14 (21.6)	0.748
PAD	176 (15.8)	73 (22.1)	0.008
CVA	93 (8.3)	26 (7.9)	0.771
Clinical presentation			
NSTEMI	698 (62.7)	238 (71.9)	0.002
STEMI	416 (37.3)	93 (28.1)	
Killip ≥ 2, *n* (%)	302 (27.1)	110 (33.2)	0.033
LVEF, %	49.5 (10.6)	48.2 (11.0)	0.057
Radial access, *n* (%)	1.042 (93.6)	314 (94.8)	0.201
Randomization, *n* (%)			
Culprit‐only	560 (50.3)	165 (49.8)	0.896
Physiology‐guided complete	554 (49.7)	166 (50.2)	
Culprit vessel, *n* (%)	*n* = 1114	*n* = 331	
Left main coronary artery	34 (3.1)	42 (12.7)	< 0.001
Left anterior descending artery	496 (44.5)	163 (49.2)	
Circumflex artery	218 (19.6)	51 (15.4)	
Right coronary artery	346 (31.1)	67 (20.2)	
Ramus intermedius artery	20 (1.8)	8 (2.4)	
Non‐culprit vessel, *n* (%)	*n* = 1443	*n* = 462	
Left anterior descending artery	468 (32.4)	119 (25.8)	0.037
Circumflex artery	463 (32.1)	167 (36.1)	
Right coronary artery	468 (32.4)	165 (35.7)	
Ramus intermedius artery	44 (3.0)	11 (2.4)	

Abbreviations: AF, atrial fibrillation; CVA, cerebrovascular accident; eGFR, glomerular filtration rate, calculated by CKD‐EPI formula; LVEF, left ventricular ejection fraction; MI, myocardial infarction; NSTEMI, non‐ST‐segment elevation myocardial infarction; PAD, peripheral artery disease; PCI, percutaneous coronary intervention; STEMI, ST‐segment elevation myocardial infarction.

## 3 Results

A total of 1445 MI patients were included, with 331 patients (793 vessels) categorized as ESV‐guided and 1114 patients (2557 vessels) as non‐ESV‐guided (Table 1). Patients in the ESV‐guided group more frequently presented with NSTEMI, higher Killip class, and a greater prevalence of left main culprit lesions. Other baseline clinical characteristics and cardiovascular risk factors were generally comparable between groups.

### 3.1 Additional Procedural Interventions Triggered by ESV Use

In the ESV‐guided group (*n* = 331), ESV prompted further optimization in 85% of procedures. Post‐dilatation with a noncompliant balloon constituted the principal maneuver (96%), predominantly via higher‐pressure inflation (67%). Balloon upsizing was less frequent, while IVUS/OCT use and additional stent implantation occurred in 3% and 1% of cases, respectively.

### 3.2 Quantitative Impact of ESV‐Guided Optimization on MLD

Across the ESV group, MLD improved from 2.51 [2.22−2.87] to 2.93 [2.41−3.32] mm after ESV‐guided adjustments and optimization (*p* < 0.001). This acute luminal gain (0.42 mm, 95% CI 0.37–0.47) reflects the mechanical effect of targeted postdilation and other corrective maneuvers performed following ESV assessment.

### 3.3 ESV and Adverse Events

Over 3 years, 381 primary endpoint events (26.4%) were recorded, including 258 deaths (17.8%), 32 strokes (2.2%), 112 MIs (7.7%), and 112 ischemia‐driven target vessel revascularizations (7.7%). The incidence of the primary endpoint was lower in the ESV‐guided group (unadjusted HR 0.75, 95% CI 0.58–0.97; *p* = 0.028) (Table 2, Figure 1). After adjustment for baseline confounders, this difference remained significant (adjusted HR 0.70, 95% CI 0.54–0.91; *p* = 0.008) (Table 2). As shown in Table 2, none of the individual components of the primary endpoint differed significantly between groups, and no differences were observed in safety outcomes.

**Table 2:** Clinical outcomes.

Outcome		No ESV (*n* = 1114)	ESV (*n* = 331)	*p*	Adjusted HR (95% CI)^a^	*p*
Primary outcome						
Composite of death, myocardial infarction, stroke, or ischemia‐driven revascularization	*n* (%)	310 (27.8)	71 (21.5)	0.028	0.70 (0.54−0.91)	0.008
	HR (95% CI)	0.75 (0.58−0.97)				
Secondary outcomes						
Cardiovascular death or myocardial infarction	*n* (%)	177 (15.9)	47 (14.2)	0.346	0.79 (0.56−1.11)	0.177
	HR (95% CI)	0.85 (0.60−1.19)				
Death	*n* (%)	206 (18.5)	52 (15.7)	0.256	0.78 (0.57−1.06)	0.115
	HR (95% CI)	0.84 (0.62−1.14)				
Cardiovascular death	*n* (%)	105 (9.4)	28 (8.5)	0.615	0.83 (0.54−1.27)	0.391
	HR (95% CI)	0.90 (0.59−1.36)				
Myocardial infarction	*n* (%)	90 (8.1)	22(6.6)	0.407	0.72 (0.40−1.29)	0.267
	HR (95% CI)	0.78 (0.44−1.40)				
Stroke	*n* (%)	30 (2.7)	2(0.6)	NA	NA	NA
	HR (95% CI)	NA				
Ischemia‐driven coronary revascularization	*n* (%)	93 (8.3)	19 (5.7)	0.153	0.59 (0.33−1.06)	0.076
	HR (95% CI)	0.66 (0.37−1.17)				
Stent thrombosis	*n* (%)	16 (1.4)	1 (0.3)	NA	NA	NA
	HR (95% CI)	NA				
Safety outcomes						
Composite of CA‐AKI, stroke, or BARC type 3−5 bleeding	*n* (%)	269 (24.1)	87 (26.3)	0.141	1.13 (0.84−1.51)	0.414
	HR (95% CI)	1.23 (0.93−1.63)				
CA‐AKI	*n* (%)	186 (16.7)	59 (17.8)	0.224	1.10 (0.77−1.57)	0.558
	HR (95% CI)	1.23 (0.88−1.73)				
BARC type 3−5 bleeding	*n* (%)	86 (7.7)	37 (11.2)	0.07	1.49 (0.93−2.39)	0.101
	HR (95% CI)	1.53 (0.97−2.43)				

Abbreviations: BARC, Bleeding Academic Research Consortium; CA‐AKI, contrast‐associated acute kidney injury.

a Adjusted hazard ratio (HR) for age, sex, diabetes mellitus, prior MI, eGFR< 60 mL/min, PAD, clinical presentation, Killip Class, and culprit vessel.

**Figure 1:**
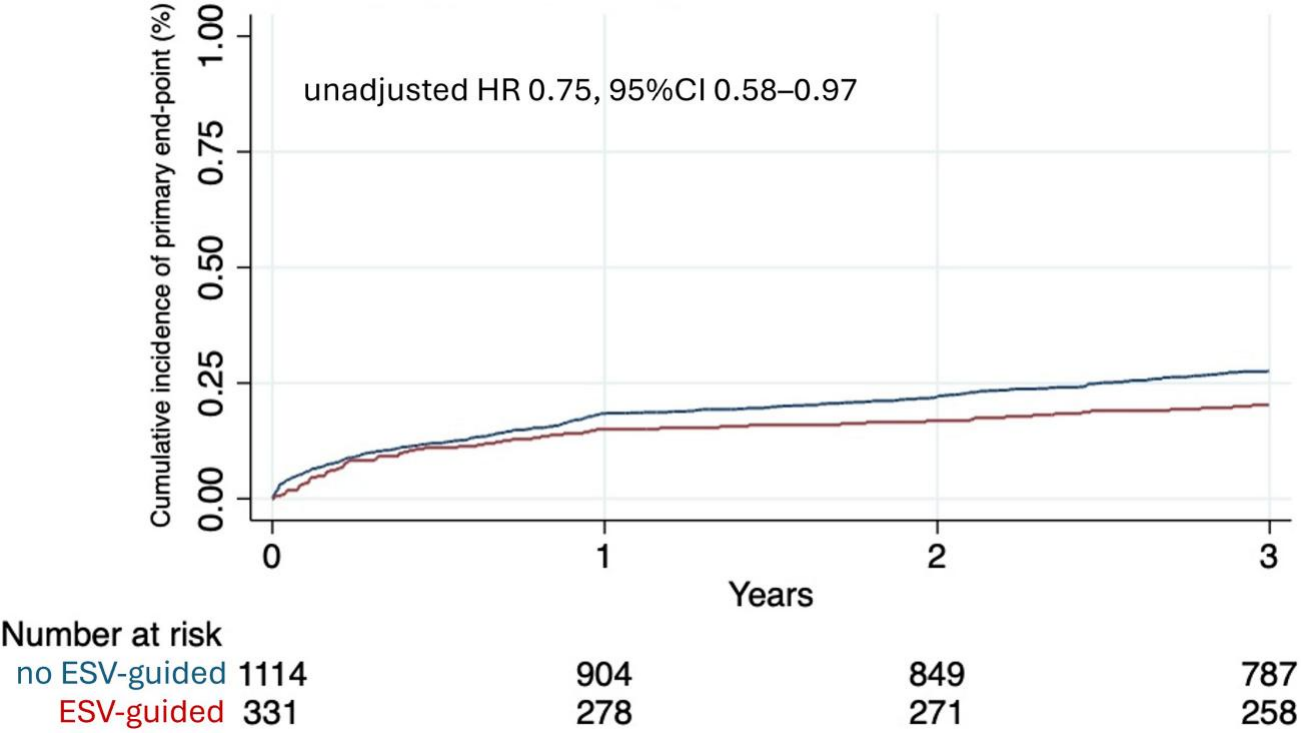
Cumulative occurrence of primary endpoint in the study groups. ESV, enhanced stent visualization. [Color figure can be viewed at wileyonlinelibrary.com]

### 3.4 Propensity‐Score Matched Analysis

In the sensitivity analysis, we used a propensity score model to perform a matched comparison. We matched the 331 ESV‐guided patients with an equal number of non‐ESV‐guided patients (Table 3). This analysis confirmed the lower incidence of the primary endpoint in the ESV‐guided group (HR 0.67; 95% CI 0.49–0.91) (Table 4, Figure 2). This difference was mainly driven by a significant reduction in ischemia‐driven coronary revascularization (HR 0.51; 95% CI 0.27–0.97) (Table 4). As expected, no differences were observed in the safety endpoints.

**Table 3:** Baseline characteristics of propensity‐score matched cohorts.

Characteristic	No ESV (*n* = 331)	ESV (*n* = 331)	*p*
Age, years	80.3 ± 3	80.6 ± 4.5	0.634
Female sex, *n* (%)	126 (38.1)	123 (37.2)	0.816
Medical history, *n* (%)			
Hypertension	267 (80.7)	282 (85.2)	0.123
Dyslipidemia	177 (53.5)	169 (51.1)	0.538
Diabetes	114 (34.4)	102 (30.8)	0.327
Current smoker	21 (6.3)	31 (9.4)	0.155
Prior MI	58 (17.5)	61 (18.4)	0.761
Prior PCI	74 (22.4)	59 (17.8)	0.155
History of AF	43 (13.0)	46 (13.9)	0.738
eGFR < 60 mL/min	57.07 (20.99)	57.14 (21.6)	0.960
PAD	79 (23.9)	73 (22.1)	0.587
CVA	26 (7.9)	26 (7.9)	0.999
Clinical presentation			
NSTEMI	236 (71.3)	238 (71.9)	0.862
STEMI	95 (28.7)	93 (28.1)	
Killip ≥ 2, *n* (%)	100 (30.2)	110 (33.2)	0.403
LVEF, %	47.93 (11.23)	48.2 (11.0)	0.744
Radial access, *n* (%)	312 (94.2)	314 (94.6)	0.813
Randomization, *n* (%)			
Culprit‐only	175 (52.9)	165 (49.8)	0.445
Physiology‐guided complete	156 (47.1)	166 (50.2)	
Culprit vessel, *n* (%)	*n* = 331	*n* = 331	
Left main coronary artery	24 (7.2)	32 (9.7)	0.169
Left anterior descending artery	177 (53.4)	163 (49.2)	
Circumflex artery	57 (17.2)	51 (15.4)	
Right coronary artery	68 (21.8)	77 (23.2)	
Ramus intermedius artery	5 (1.5)	8 (2.4)	
Non‐culprit vessel, *n* (%)	*n* = 442	*n* = 462	
Left anterior descending artery	104 (24.4)	119 (26.0)	0.393
Circumflex artery	140 (33.2)	165 (36.0)	
Right coronary artery	162 (38.4)	163 (35.6)	
Ramus intermedius artery	17 (4.0)	11 (2.4)	

Abbreviations: AF, atrial fibrillation; CVA, cerebrovascular accident; eGFR, glomerular filtration rate, calculated by CKD‐EPI formula; LVEF, left ventricular ejection fraction; MI, myocardial infarction; NSTEMI, non‐ST‐segment elevation myocardial infarction; PAD, peripheral artery disease; PCI, percutaneous coronary intervention; STEMI, ST‐segment elevation myocardial infarction.

**Table 4:** Clinical outcomes of the propensity‐score matched cohorts.

Outcome		No ESV (*n* = 331)	ESV (*n* = 331)	*p*
Primary outcome				
Composite of death, myocardial infarction, stroke, or ischemia‐driven revascularization	*n* (%)	101 (30.5)	71 (21.5)	0.008
	HR (95% CI)	0.67 (0.49−0.91)		
Secondary outcomes				
Cardiovascular death or myocardial infarction	*n* (%)	64 (19.3)	47 (14.2)	0.077
	HR (95% CI)	0.71 (0.48−1.06)		
Death	*n* (%)	66 (19.9)	52 (15.7)	0.166
	HR (95% CI)	0.77 (0.54−1.11)		
Cardiovascular death	no. (%)	38 (11.5)	28 (8.5)	0.199
	HR (95% CI)	0.73 (0.45−1.18)		
Myocardial infarction	*n* (%)	32 (9.7)	22 (6.6)	0.168
	HR (95% CI)	0.69 (0.35−1.37)		
Stroke	*n* (%)	10 (3)	2 (0.6)	NA
	HR (95% CI)	NA		
Ischemia‐driven coronary revascularization	*n* (%)	36 (10.9)	19 (5.7)	0.041
	HR (95% CI)	0.51 (0.27−0.97)		
Stent thrombosis	*n* (%)	7 (2.1)	1 (0.3)	NA
	HR (95% CI)	NA		
Safety outcomes				
Composite of CA‐AKI, stroke, or BARC type 3−5 bleeding	*n* (%)	94 (28.4)	87 (26.3)	0.543
	HR (95% CI)	1.16 (0.81−1.64)		
CA‐AKI	*n* (%)	67 (20.2)	59 (17.8)	0.434
	HR (95% CI)	1.13 (0.74−1.73)		
BARC type 3−5 bleeding	*n* (%)	28 (8.5)	37 (11.2)	0.241
	HR (95% CI)	1.73 (0.91−3.26)		

Abbreviations: BARC, Bleeding Academic Research Consortium; CA‐AKI, contrast‐associated acute kidney injury.

**Figure 2:**
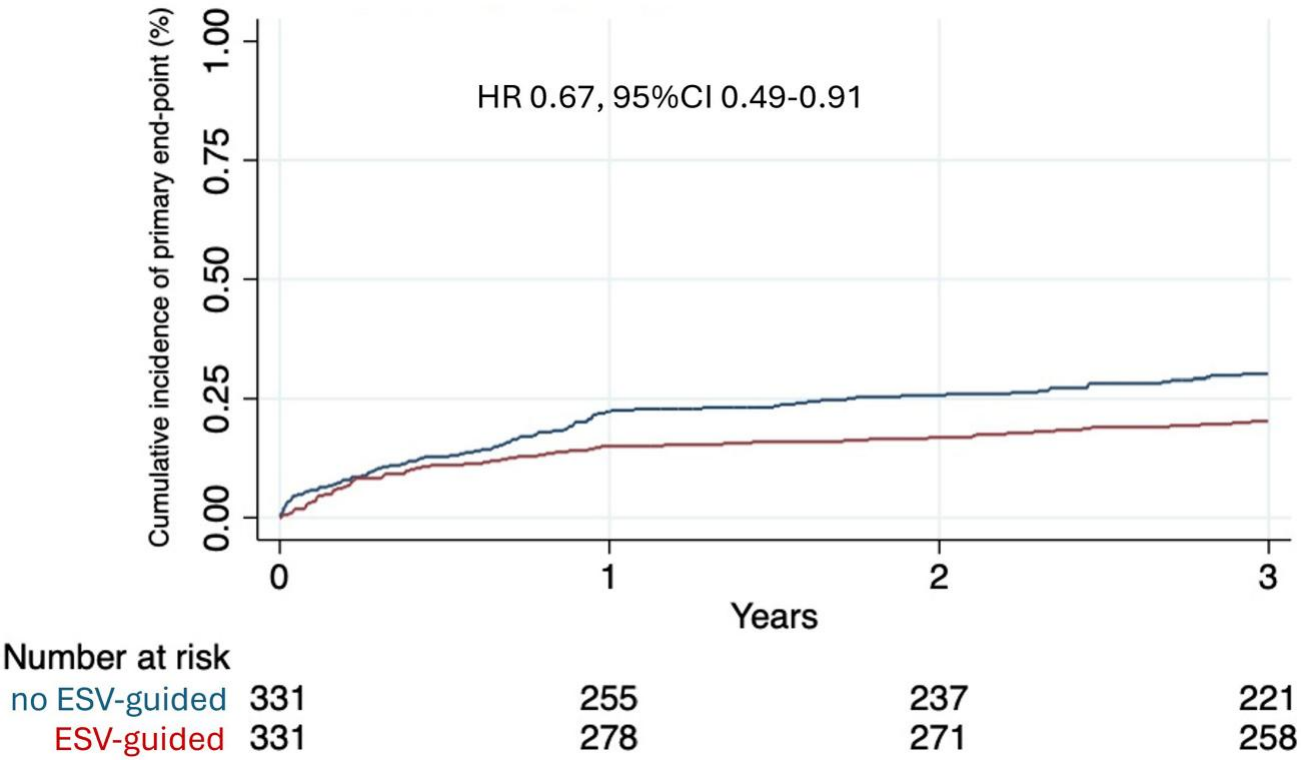
Cumulative occurrence of primary endpoint in the propensity‐score matched subgroups. ESV, enhanced stent visualization. [Color figure can be viewed at wileyonlinelibrary.com]

## 4 Discussion

In this prespecified analysis of the FIRE trial, we evaluated the role of ESV during PCI in older patients with MI and multivessel disease. In this high‐risk population, ESV use was associated with a high rate of operator‐driven optimization, a measurable acute luminal gain, and a lower incidence of long‐term adverse events. Although ESV does not provide the detailed anatomical information obtainable with IVUS or OCT, our findings suggest that it may serve as a useful adjunct for improving stent deployment quality in settings where intravascular imaging is seldom used. Optimal stent expansion is a cornerstone of durable PCI success [7, 8, 9]. Underexpansion, malapposition, and geographic miss are well‐established predictors of stent failure, contributing to restenosis, thrombosis, and recurrent ischemia [7, 8, 9]. While IVUS and OCT offer unparalleled resolution for assessing stent architecture and lesion morphology, their adoption in routine clinical practice remains limited, particularly in older patients, due to concerns related to procedural complexity, time requirements, contrast volume, and cost. As a result, most stent optimizations still rely on angiography alone, despite its known inability to detect subtle mechanical issues. ESV technologies, such as ClearStent, were developed to partially bridge this gap by enhancing stent visibility without adding procedural steps [1, 2, 3, 4]. Through motion‐correction algorithms and real‐time image enhancement, ESV improves the delineation of stent contour and allows operators to recognize gross underexpansion or geographic miss that may be missed on standard angiography [1, 2, 3, 4]. Although it cannot quantify minimal stent area or characterize plaque, ESV provides actionable information that can prompt additional optimization or selective use of advanced imaging [1, 2, 3, 4].

In our analysis, ESV triggered further operator‐driven refinement in the majority of cases (85%). Most interventions involved additional postdilation with noncompliant balloons, using either higher inflation pressures or larger diameters, and these maneuvers produced a significant acute luminal gain (median +0.42 mm). The specific morphological reason for each corrective maneuver was not prospectively recorded. While modest in absolute terms, such improvements are consistent with established evidence showing the prognostic benefit of achieving adequate stent expansion. Importantly, these corrective actions were performed without increasing procedural complications. The FIRE population reflects real‐world older patients (≥ 75 years) presenting with MI and multivessel disease, a group characterized by extensive calcification, diffuse atherosclerosis, and challenging anatomy [17]. Ironically, these patients are those who would benefit most from imaging‐guided PCI, yet they are the least likely to receive it. In FIRE, IVUS/OCT use was < 5%, mirroring contemporary registries [18]. Within this context, ESV emerges as a practical and feasible adjunct. By enhancing angiographic interpretation, ESV provides an intermediate step between pure angiography‐guided PCI and full imaging‐guided optimization—an approach that may be particularly valuable in older or frail individuals where procedural simplicity is essential. Clinically, ESV use was associated with a significantly lower incidence of the composite primary endpoint at 3 years, and this benefit persisted after comprehensive adjustment. Although this observational analysis cannot infer causality, the findings are biologically plausible. Better acute stent expansion likely facilitates more favorable vessel healing, reduces neointimal proliferation, and lowers the risk of stent failure. Indeed, even small improvements in lumen dimensions can meaningfully reduce shear stress and thrombogenic potential, supporting the mechanistic link between ESV‐guided optimization and clinical benefit.

### 4.1 Limitations

Several limitations merit consideration. First, ESV use was not randomized and was left to operator discretion, introducing the possibility of selection bias. Because ESV use was not mandated by the trial protocol, acquisition in two orthogonal projections was not systematically required or prospectively recorded. In addition, ESV provides a projection‐dependent two‐dimensional representation of a three‐dimensional stent; orthogonal acquisitions were not standardized, and underexpansion outside the selected projection may therefore have remained undetected. Although multivariable and propensity‐score adjustments were applied, residual confounding cannot be excluded. Second, ESV offers only a qualitative assessment of stent expansion and lacks the quantitative data provided by IVUS or OCT. The observed increase represents an angiographic QCA estimate and should not be interpreted as a direct measurement of minimal stent area or three‐dimensional stent expansion, which requires IVUS or OCT. Third, the observed acute luminal gain, while statistically significant, is modest, and its direct relationship with long‐term healing remains to be fully determined. Because the operator‐reported morphological cause prompting post‐dilatation was not prospectively collected, the relative contributions of focal or diffuse underexpansion, edge abnormalities, and geographic miss could not be separately quantified. Finally, the study was not powered to detect differences in individual clinical endpoints, which should be interpreted with caution.

## 5 Conclusions

In older MI patients with multivessel disease, ESV use was associated with more frequent procedural optimization, improved acute stent expansion, and lower long‐term adverse events. Although ESV cannot replace intravascular imaging, it represents a practical and effective adjunct for enhancing stent deployment in real‐world settings where imaging remains underused. Wider adoption of ESV may help reduce the gap between angiography‐guided and imaging‐guided PCI, ultimately contributing to safer and more effective revascularization in this vulnerable population.

## Ethics Statement

All patients provided written informed consent, and the protocol was approved by the institutional review board at each participating center.

## Conflicts of Interest

S.B. received a speaker's fee, a research grant, and performed advisory board activity for Siemens Healthineers. The other authors declare no conflicts of interest.

## Data Availability

Data supporting this manuscript are available from the FIRE Study Executive Committee upon reasonable request.
